# National and Regional Trends in Police Pursuit Fatalities in the US

**DOI:** 10.1001/jamanetworkopen.2024.46415

**Published:** 2024-11-21

**Authors:** Jemar R. Bather, Melody S. Goodman

**Affiliations:** 1 Center for Anti-racism, Social Justice & Public Health, New York University School of Global Public Health, New York; 2 Department of Biostatistics, New York University School of Global Public Health, New York

## Abstract

This cross-sectional study examines rates of police pursuit fatalities in the US from 2017 to 2021.

## Introduction

Research spanning from 1982 to 2020 suggests an increasing trend in fatalities resulting from police pursuits in the US.^1,2,3,4^ To address this critical public health issue, the US Congress tasked the Police Executive Research Forum with providing police pursuit policy recommendations for all US law enforcement agencies.^4^ Currently, each law enforcement agency develops and implements its own police pursuit policy, with some being more restrictive than others.^2,4^ Prior national reports on police pursuit fatalities in the US are limited in 2 aspects: (1) they did not include information from supplemental sources such as public court cases and media outlets, and (2) most prior examinations report pooled estimates, lacking regional estimates by year.^1,3,4,5^

## Methods

This cross-sectional study was deemed exempt from review by the New York University institutional review board and did not require informed consent because the data used in this study are deidentified, do not contain information about living individuals, and are publicly available. We analyzed data from the Fatal Police Pursuits Database compiled by investigative reporters at the *San Francisco Chronicle*, which catalogs records of fatal police pursuits in the US (excluding Puerto Rico) from 2017 to 2022.^6^ Of the 3336 fatal police pursuits recorded in the Fatal Police Pursuits Database, 332 (10.0%) that occurred in 2022 were excluded due to incomplete data, resulting in the analytic sample (N = 3004). Investigative reporters measured the total fatality count as the number of confirmed deaths resulting from the police pursuit.^6^ We computed the total fatality rate as the total fatality count divided by the number of people in the population using annual population estimates and region categories from the US Census Bureau. Joinpoint models estimated the average annual percent change (AAPC) in fatality rates and corresponding 95% CIs for the US overall and stratified by region from 2017 to 2021. Statistical significance was assessed as 2-sided *P* < .05. Statistical analyses were performed using the Joinpoint Trend Analysis Software version 5.2.0 (Statistical Methodology and Applications Branch, Surveillance Research Program, National Cancer Institute). Additional methods are described in the eMethods of Supplement 1.

## Results

The Table and Figure illustrate trends in police pursuit fatalities overall and stratified by region from 2017 to 2021. During this period, the US experienced 4415 police pursuit fatalities, with a statistically significant increasing trend (2017-2021 AAPC, 10.13 [95% CI, 7.46 to 12.32]). The South region had 2242 fatalities, showing a significant upward trend (AAPC, 17.51 [95% CI, 9.43 to 25.12]). However, we did not observe statistically significant trends in the Midwest (1045 fatalities; AAPC, −6.54 [95% CI, −17.10 to 3.51]), West (843 fatalities; AAPC, 15.27 [95% CI, −2.85 to 35.00]), or Northeast (285 fatalities; AAPC, 3.61 [95% CI, −4.07 to 10.96]) regions.

**Table.** Estimated Trends in Police Pursuit Fatalities in the US Overall and by Census Region, Fatal Police Pursuits Database, 2017-2021

Location	2017-2021		
	Total fatalities, No.	AAPC (95% CI)	*P* value
United States	4415	10.13 (7.46 to 12.32)	<.001
Region			
South	2242	17.51 (9.43 to 25.12)	<.001
Midwest	1045	−6.54 (−17.10 to 3.51)	.15
West	843	15.27 (−2.85 to 35.00)	.11
Northeast	285	3.61 (−4.07 to 10.96)	.40

Abbreviation: AAPC, average annual percent change.

**Figure.**
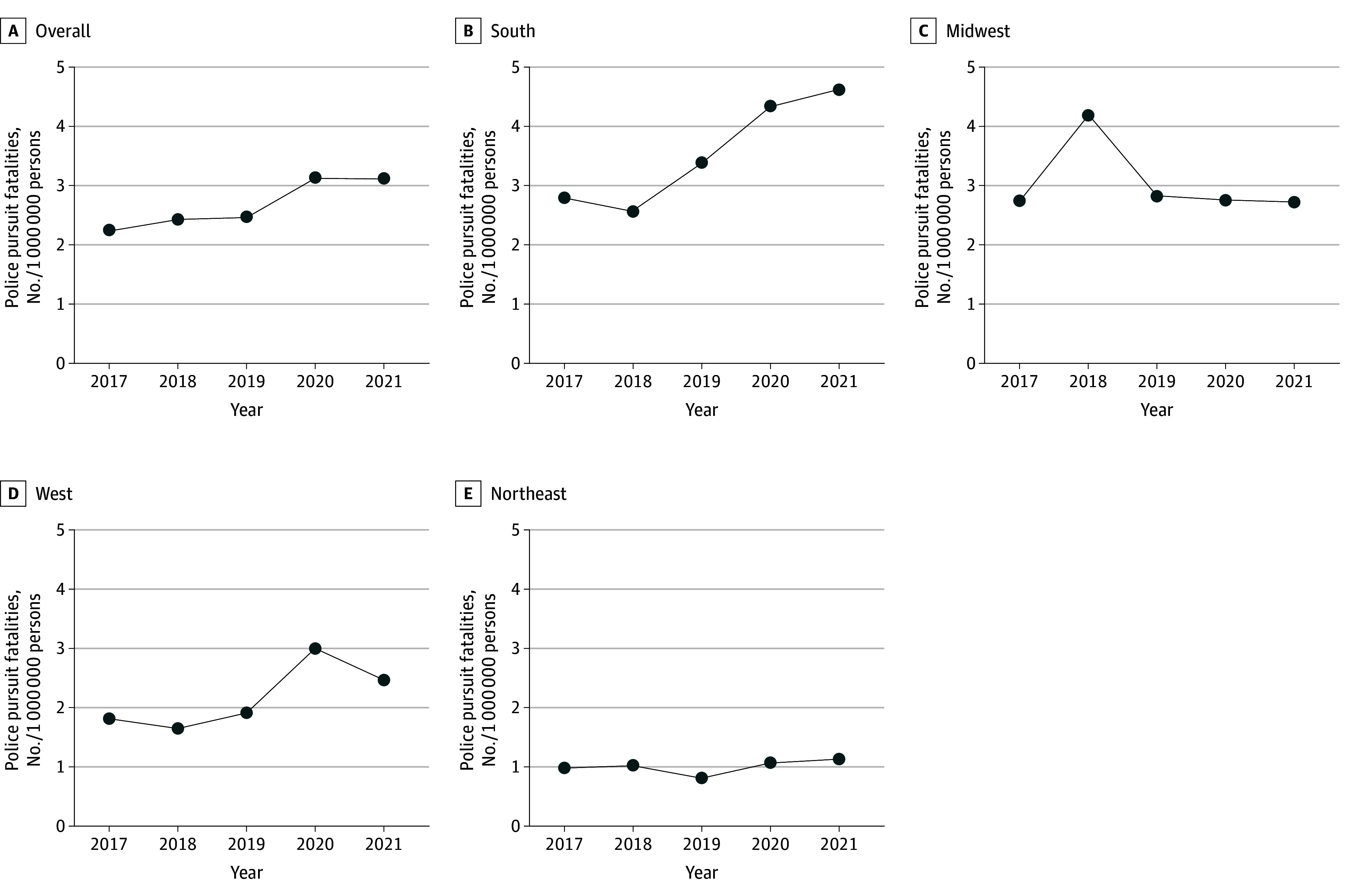
Observed Trends in Police Pursuit Fatalities in the US Overall and by Census Region, Fatal Police Pursuits Database, 2017-2021

## Discussion

Drawing on a novel data source, we provided national and regional estimates of police pursuit fatalities in the US from 2017 to 2021. Although the Fatal Police Pursuits Database is innovative (eg, including data from public court cases), it requires validation. Future studies should link the Fatal Police Pursuits Database to other data sources to characterize its robustness, representativeness, and generalizability and identify potential gaps in its coverage.

This study has several limitations. The estimates presented in this analysis may be underreported and susceptible to measurement error.^4^ The dataset did not allow us to provide passenger-specific temporal fatality trends or quantify the total number of fatalities per pursuit. The analysis included a short time series, warranting future investigations that use more periods. We could not characterize the reason for the initial pursuit and each individual’s role. The Fatal Police Pursuits Database did not include data from Puerto Rico. Future research could address these limitations by incorporating more detailed sociodemographic data, expanding the analysis time frame, and including data from Puerto Rico and other jurisdictions. Such efforts would further enhance our understanding of this critical public health issue, inform evidence-based policy decisions, and potentially reduce police pursuit fatalities.

## Notes

Supplement 1.
eMethods

eReferences

Supplement 2.
Data Sharing Statement
