# Supplementary material for: National and Regional Trends in Police Pursuit Fatalities in the US
Source: JAMA Netw Open. 2024 Nov 21;7(11):e2446415. doi: 10.1001/jamanetworkopen.2024.46415 (PMC11582929; doi:10.1001/jamanetworkopen.2024.46415)
Supplement: Supplement 1. — eMethods eReferences [file jamanetwopen-e2446415-s001.pdf]

Supplemental Online Content

Bather JR, Goodman MS. National and regional trends in police pursuit fatalities in the US. *JAMA Netw Open.* 2024;7(11):e2446415. doi:10.1001/jamanetworkopen.2024.46415

eMethods
eReferences

This supplemental material has been provided by the authors to give readers additional information about their work.

© 2024 Bather JR et al. *JAMA Network Open*.

eMethods

Study Design and Setting

We analyzed data from the Fatal Police Pursuits Database, which catalogs records of fatal police pursuits in the US (excluding Puerto Rico) from 2017 to 2022.[1] Compiled by investigative reporters at the San Francisco Chronicle,[1] this database draws information from the National Highway Traffic Safety Administration, public court cases, media outlets, and data repositories constructed by other research groups. Further details about the data collection procedures are described elsewhere.[1] The New York University Institutional Review Board considered this secondary data analysis exempt since the data used in this study are de-identified, do not contain information about living individuals, and are publicly available (https://github.com/sfchronicle/police_pursuits). We adhered to the Declaration of Helsinki and Strengthening the Reporting of Observational Studies in Epidemiology guidelines.

Analytic Sample

A total of 3,336 fatal police pursuits were recorded in the Fatal Police Pursuits Database. We excluded 332 (10%) fatal police pursuits that occurred in 2022 because the investigative reporters noted that the 2022 data was incomplete when the Fatal Police Pursuits Database was released. The final analytic sample comprised 3,004 fatal police pursuits.

Total Fatality Rate

Investigative reporters measured the total fatality count as the number of confirmed deaths resulting from the police pursuit.[1] We computed the total fatality rate as the total fatality

© 2024 Bather JR et al. *JAMA Network Open*.

count divided by the number of people in the population. We used annual population estimates and region categories from the US Census Bureau.[2–4] We multiplied the total fatality rate by one million for interpretability (fatalities per 1,000,000 persons).

Statistical Analysis

Per Kim et al.,[5] we fit the following joinpoint regression model to analyze fatality rate trends from 2017 to 2021:

$$\log(\mu) = \beta_0 + \beta_1 \mathrm{X},$$

where μ denoted the expected fatality rate given the number of years over the study period (X), and β_1 estimated the association (slope) of time on fatality rates. We derived the average annual percent change (AAPC) as $(exp^{\beta} - 1) \times 100$, which described the average rate at which fatality rates changed per year over the study period. Joinpoint models estimated the AAPC in fatality rates and corresponding 95% confidence intervals (CIs) for the US (excluding Puerto Rico) and each US census region. We did not test for statistically significant change points due to the recommendation of needing at least seven time periods to test for change points.[6] Statistical significance was assessed as two-sided $P<0.05$. We used the permutation method for model selection, which applied the Bonferroni correction to address multiplicity.[5] Statistical analyses were performed using the Joinpoint Trend Analysis Software (Version 5.2.0).[7] The results from this analysis were not intended to extrapolate or predict trends outside of the study period.

© 2024 Bather JR et al. *JAMA Network Open*.

eReferences

1. Gollan J, Neilson S. Fatal Police Pursuits Database. San Francisco Chronicle: Code from the San Francisco Chronicle Interactive Team. March 11, 2024. Accessed May 17, 2024. https://github.com/sfchronicle/police_pursuits

2. US Census Bureau. State Population Totals and Components of Change: 2010-2019. Census.gov. 2021. Accessed July 21, 2024. https://www.census.gov/data/tables/time-series/demo/popest/2010s-state-total.html

3. US Census Bureau UC. State Population Totals and Components of Change: 2020-2023. Census.gov. 2024. Accessed July 21, 2024. https://www.census.gov/data/tables/time-series/demo/popest/2020s-state-total.html

4. US Census Bureau UC. Geographic Levels. Census.gov. 2021. Accessed July 21, 2024. https://www.census.gov/programs-surveys/economic-census/guidance-geographies/levels.html

5. Kim HJ, Fay MP, Feuer EJ, Midthune DN. Permutation Tests for Joinpoint Regression with Applications to Cancer Rates. *Stat Med*. 2000;19(3):335-351. doi:10.1002/(sici)1097-0258(20000215)19:3<335::aid-sim336>3.0.co;2-z

6. National Cancer Institute. Number of Joinpoints. Joinpoint Help System. 2024. Accessed July 5, 2024. https://surveillance.cancer.gov/help/joinpoint/setting-parameters/method-and-parameters-tab/number-of-joinpoints

7. National Cancer Institute. Joinpoint Regression Program. Joinpoint Trend Analysis Software. 2024. Accessed July 5, 2024. https://surveillance.cancer.gov/joinpoint/

© 2024 Bather JR et al. *JAMA Network Open*.
