# Supplementary material for: National and Regional Trends in Police Pursuit Fatalities in the US
Source: JAMA Netw Open. 2024 Nov 21;7(11):e2446415. doi: 10.1001/jamanetworkopen.2024.46415 (PMC11582929; doi:10.1001/jamanetworkopen.2024.46415)
Supplement: Supplement 2. — Data Sharing Statement [file jamanetwopen-e2446415-s002.pdf]

Data Sharing Statement

Bather. National and Regional Trends in Police Pursuit Fatalities in the US. *JAMA Netw Open*. Published November 21, 2024. doi:10.1001/jamanetworkopen.2024.46415

Data
Data available: Yes
Data types: Other (please specify)
Additional Information: The data that support the findings of this study are openly available (https://github.com/sfchronicle/police_pursuits).
How to access data: The data that support the findings of this study are openly available (https://github.com/sfchronicle/police_pursuits).
When available: With publication

Supporting Documents
Document types: None

Additional Information
Who can access the data: Anyone requesting the data
Types of analyses: Any purpose
Mechanisms of data availability: Publicly available
